# The effect of SOX4 gene 3′UTR polymorphisms on osteoporosis

**DOI:** 10.1186/s13018-021-02454-x

**Published:** 2021-05-18

**Authors:** Guo Li, Zuchao Gu, Yue He, Chongwen Wang, JiQiang Duan

**Affiliations:** 1 Department of Orthopedics, Sichuan Chengdu First People’s Hospital, 18 Wanxiang North Road, Chengdu, 610041 Sichuan China; 2 Department of Orthopedics, Zibo Central Hospital, 54 West Gongqingtuan Road, Zibo, 255036 Shandong China

**Keywords:** Osteoporosis, SRY-related high-mobility-group box gene 4, Single nucleotide polymorphism, Gene-environment interaction

## Abstract

**Objective:**

This study aimed to explore the correlation between the SRY-related high-mobility-group box gene 4 (SOX4) 3′ untranslated region (UTR) single nucleotide polymorphism (SNP) and osteoporosis susceptibility.

**Methods:**

The study recruited 330 osteoporosis patients (the case group) and 330 non-osteoporosis patients (the control group) in Sichuan Chengdu First People’s Hospital and Zibo Central Hospital from August 2016 to August 2019. Sanger sequencing was used to analyze the genotypes of SOX4 gene rs79958549, rs139085828, and rs201335371 loci. Multi-factor dimensionality reduction (MDR) was used to analyze the interaction between the SOX4 gene rs79958549, rs139085828, and rs201335371 loci and the clinical characteristics of the subjects.

**Results:**

The risk of osteoporosis in the carriers of A allele at SOX4 rs79958549 was 5.40 times that in the carriers of the G allele (95% CI 3.25–8.96, *P* < 0.01). The risk of osteoporosis in the carriers of the A allele at SOX4 rs139085828 was 1.68 times that in the carriers of the G allele (95% CI 1.45–1.85, *P* < 0.01). The risk of osteoporosis in the carriers of the T allele at SOX4 rs201335371 was 0.54 times that in the carriers of the C allele (95% CI 0.43–0.69, *P* < 0.01). The SOX4 gene rs79958549, rs139085828, and rs201335371 A-A-C haplotype (OR = 5.14, 95% CI 2.45–10.57, *P* < 0.01) were associated with increased risk of osteoporosis and G-G-T haplotype was significantly associated with decreased risk of osteoporosis (OR = 0.48, 95% CI 0.38–0.62, *P* < 0.01). The interaction among the factors of sex, smoking, drinking, rs79958549, rs201335371 was the best model for osteoporosis prediction, and the risk for osteoporosis in ‘high-risk combination’ was 2.74 times that of ‘low-risk combination’ (95% CI 1.01–7.43, *P* = 0.04). Multiple logistic regression analysis revealed that the risk factors for osteoporosis were BMD (OR = 5.85, 95% CI 2.88–8.94, *P* < 0.01), *T* score (OR = 8.54, 95% CI 5.66–10.49, *P* < 0.01), *Z* score (OR = 3.77, 95% CI 2.15–8.50, *P* < 0.01), rs79958549 SNP (OR = 6.92, 95% CI 3.58–8.93, *P* < 0.01), and rs139085828 SNP (OR = 2.36, 95% CI 1.85–4.27, *P* < 0.01). The protective factor for osteoporosis was rs201335371SNP (OR = 0.48, 95% CI 0.32–0.75, *P* < 0.01).

**Conclusion:**

The SOX4 gene SNPs rs79958549, rs139085828, and rs201335371 loci were significantly associated with osteoporosis risk.

## Introduction

Osteoporosis, commonly seen in the elderly population, is a disease characterized by an imbalance in bone homeostasis involving multiple organs, which can lead to a reduction in bone weight-bearing capacity and an increase in bone fragility and easiness to multi-site fractures [1–3]. The main pathogenesis of osteoporosis is the imbalance of osteoblastic bone formation and osteoclastic bone resorption resulting in the reduction of bone mass and the destruction of bone microstructure, thus increasing bone fragility and decreasing bone strength [4, 5]. The body strictly regulates the activation, differentiation, and apoptosis of osteoblasts and osteoclasts to maintain the dynamic balance of bone formation and bone resorption, in which genetic factors play an important role [6, 7].

In the 1990s, the discovery of the male sex determining region Y (SRY) led to the discovery of the entire family of key regulatory genes SOX [8]. The transcription factors SOX proteins encoded by SOX genes control the fate of cells in many lineages. These transcription factors can promote the development of key systems such as the cardiovascular system, central and peripheral nervous system, endocrine system, and skeletal system. There are 20 SOX genes in the mammalian genome, among which SOXC genes (including SOX4, SOX11, and SOX12 genes) are related to bone development.

The SOX4 gene is located at 6p22.3 and contains 4879bp, but only has one exon encoded as the SOX4 protein. This gene is an important member of the SOX family and participates in the regulation of embryo development and differentiation by encoding transcription factors [3, 9]. The mutation, deletion or overexpression of the SOX4 gene not only can cause dysplasia or congenital diseases [10] but also are closely related to the formation and development of tumors [11].

Nissen-Meyer et al. [12] found that SOX4+/− heterozygote knockout mice suffered from osteoporosis in both young and adult. Compared with the control group, the proliferation and differentiation of the osteoblast progenitor cells in these mice were delayed, the bone cortex and trabecular bone were thinner, and the bone formation rate reduced; however, the bone cell rate was normal. In addition, some researchers have found that SNPs around the SOX4 gene sequence are related to the total hip bone mineral density [13].

In this study, we selected the SNP loci with a minor allele frequency (MAF)> 0.01 in the 3′UTR region of the SOX4 gene. The SNP loci chosen were rs79958549, rs139085828, and rs201335371, which have received no attention in the studies so far. As the population with these SNP loci is relatively large, the study on the genetic background of this population and its association with osteoporosis is of great significance for the prevention and treatment of osteoporosis.

## Materials and methods

### Subjects

From August 2016 to August 2019, 330 patients with osteoporosis were selected as the case group, including 165 men and 165 women, aged from 47 to 89, with the average age of (65.80 ± 10.71). In the same period, 330 non-osteoporosis patients were selected as the control group in a 1:1 ratio according to the age, gender, and BMI of the case group, including 171 males and 159 females, aged from 48 to 87, with the average age of (64.95 ± 8.88). Diagnostic criteria for osteoporosis included the following: (1) fragile fracture of the hip or vertebral body; (2) *T*-score of lumbar spine 1-4 (L1-4) bone mineral density (BMD) measured by dual-energy X-ray absorptiometry (DXA) was <− 2.5; (3) BMD tests were consistent with low bone mass (− 2.5<*T*-score<− 1.0) + fragile fracture of the proximal humerus, pelvis, or distal forearm. The criteria for the control group included the following: (1) BMD *T*-score ≥− 1.0; (2) BMD tests were consistent with low bone mass (− 2.5<*T*-score<− 1.0) without fragile fracture risk. Inclusion criteria were as follows: (1) the anatomical structure of the lumbar spine suitable for DXA measurement, no serious scoliosis deformity, and no screw rod and other internal fixators placed in the lumbar vertebra; (2) BMI ≥ 18.5 kg/m^2^; (3) good health, able to stand or move regularly for at least 30 min a day. Exclusion criteria were as follows: (1) patients with diseases known to affect bone metabolism, such as severe malabsorption syndrome, chronic liver disease, inflammatory bowel disease, primary hyperparathyroidism that are not effectively controlled, hypercalcemia, Paget’s bone disease, active kidney stones, osteogenesis imperfecta, and pituitary disease; (2) patients with secondary osteoporosis, such as rheumatoid arthritis, osteomalacia, multiple myeloma, and gout; (3) patients who have taken fluoride preparations continuously in the past 2 years; (4) patients who have been continuously treated with bisphosphonates or PTH for more than 15 days within 1 year; (5) patients who have continuously used estrogen receptors modulators within 6 months; (6) patients who have continuously received calcitonin, estrogen, corticosteroids, calcitriol, and other drugs that can change bone metabolism within 3 months; (7) patients with severe liver and kidney diseases, peptic ulcer, rheumatic and immune diseases, malignant tumors, and other serious underlying diseases; (8) patients with factors that affect the measurement results of BMD, such as the history of lumbar spine fixation surgery and ankylosing spondylitis. This study was approved by the Medical Ethics Committee of Sichuan Chengdu First People’s Hospital and Zibo Central Hospital. All subjects have signed informed consent.

### Genotyping

Genomic DNA was extracted from the peripheral blood of the subjects using the DNeasy Blood & Tissue Kit (Qiagen, Valencia, CA) and stored in the refrigerator at – 80 °C. The extracted genomic DNA was used as a template for the PCR amplification, thereby obtaining DNA fragments containing SOX4 gene rs79958549, rs139085828, and rs201335371. The sequences of primer for PCR amplification were as follows: rs79958549 Forward primer: 5′-CGC AGG CAG GGA GAA GG-3′, Reverse primer: 5′-TTG ATC CGA CGA CGA GAA CG-3′; rs139085828 Forward primer: 5′-CCG AGA ACC CCG TTG GAA G-3′; Reverse primer: 5′-TCA GTT TGA CCG TGA ACC CC-3′; rs201335371 Forward primer: 5′-GGA CGT ATT TAT ACT GGC CAA ACA-3′, Reverse primer: 5′-AGA CGT AAA ATG GCG TGG GT-3′. The PCR amplification reaction included the following: 2.0 μl 10 × buffer, 10 pmol/L Forward primer, 10 pmol/L Reverse primer, 200 μmol/L dNTP, 1.5 mmol/L Mg2+, 2.5 U Taq enzyme. The PCR reaction conditions included the following: 95 °C for 5 min; 30 cycles of 95 °C for 20 s; 58 °C for 20 s, 72 °C for 20 s, and at last 72 °C extension for 10 min. PCR products were analyzed by Sanger sequencing and then compared with the sequences data in the dbSNP database (https://www.ncbi.nlm.nih.gov/snp/). The genotypes of the subjects were determined according to the matching results.

### Statistical analysis

The *χ*^2^ test was used to assess whether the genotype frequencies of SOX4 gene rs79958549, rs139085828, and rs201335371 conformed to Hardy Weinberg equilibrium. The *χ*^2^ test was used for the statistical analysis of the categorical variables [*n*(%)]. The *t* test or one-way analysis of variance was used for the statistical analysis of continuous variables (mean ± SD). Logistic regression was performed to analyze the correlation between the genotypes of SOX4 gene rs79958549, rs139085828, and rs201335371 and osteoporosis risk and calculate the odds ratio (OR) and 95% confidence interval (CI) with the adjustment for age, gender, BMI, smoking, and drinking. Multi-factor dimensionality reduction (MDR) was used to analyze the interaction between SOX4 gene rs79958549, rs139085828, and rs201335371 locus alleles and subjects’ clinical characteristics of age, sex, BMI, smoking, and alcohol consumption. The best model *P* < 0.05 indicates that there is an interaction between gene polymorphisms, and permutation test: *P* < 0.05 verified statistical significance. Consistency tests indicate the degree of statistical conformity, with 10 being a perfect match. SPSS 20.0 (SPSS, Chicago, IL, USA) was employed for statistical analysis. All statistical tests were two-sided, and the level of statistical significance was set at *P* value < 0.05.

## Results

### Clinical characteristics

The clinical characteristics of 330 osteoporosis patients (case group) and 330 controls selected in this study are shown in Table 1. There were no statistically significant differences in the clinical data of age, gender, body mass index (BMI), smoking, and drinking between the case and the control group (*P* > 0.05). The bone mineral density (BMD), *T*-score, and *Z*-score of the case group were significantly lower than those of the control group, and the differences were statistically significant (*P* < 0.01).

**Table 1:** Comparison of clinical characteristics between the osteoporosis patients and the control group

	Case(*n* = 330)	Control(*n* = 330)	*P*
Age(years, mean ± SD)	65.80 ± 10.71	64.95 ± 8.88	0.27
Gender[n(%)]			0.64
Male	165(50.00%)	171(51.82%)	
Female	165(50.00%)	159(48.18%)	
BMI(kg/m^2^, mean ± SD)	22.77 ± 2.31	22.68 ± 2.12	0.60
Smoking			0.51
Yes	117(35.45%)	109(33.03%)	
No	213(64.55%)	221(66.97%)	
Drinking			0.69
Yes	126(38.18%)	121(36.67%)	
No	204(61.82%)	209(63.33%)	
BMD(g/m^2^)	0.72 ± 0.14	1.02 ± 0.21	< 0.01
*T*-score	− 3.29 ± 0.75	0.25 ± 0.17	< 0.01
*Z*-score	− 3.38 ± 1.33	− 1.25 ± 0.51	< 0.01

*SD* standard deviation, *BMI* body mass index, *BMD* bone mineral density

### Association of SOX4 gene polymorphisms with susceptibility to osteoporosis

The genotypes and allele frequencies of SOX4 gene rs79958549, rs139085828, and rs201335371 are shown in Table 2. The analysis results indicated that SOX4 gene rs79958549, rs139085828, and rs201335371 genotype frequencies of the controls were in Hardy-Weinberg equilibrium (*P* > 0.05). When the GG genotype of SOX4 gene rs79958549 as a reference, the GA genotype, AA genotype, dominant model, and recessive model were all associated with an increased risk of osteoporosis (*P* < 0.05), and there was no significant correlation between the additive model and the risk of osteoporosis (*P* = 0.17). The risk of osteoporosis in the A allele carriers was 5.40 times that in the G allele carriers (95% CI 3.25–8.96, *P* < 0.01). When the GG genotype of SOX4 gene rs139085828 as a reference, the GA genotype, AA genotype, dominant model, and recessive model were all associated with an increased risk of osteoporosis (*P* < 0.05) and there was no significant correlation between the additive model and the risk of osteoporosis (*P* = 0.36). The risk of osteoporosis in the A allele carriers was 1.68 times that in the G allele carriers (95% CI 1.45–1.85, *P* < 0.01). When the CC genotype of SOX4 gene rs201335371 as a reference, the CT genotype, TT genotype, dominant model, recessive model, and additive model were associated with a reduced risk of osteoporosis (*P* < 0.05) and the risk of osteoporosis in the T allele carriers was 0.54 times that in the C allele carriers (95% CI 0.43–0.69, *P* < 0.01).

**Table 2:** The genotypes and allele frequencies of SOX4 gene rs79958549, rs139085828, rs201335371, and osteoporosis susceptibility

	Case(*n* = 330)	Control(*n* = 330)	HWE *P*	OR (95% CI)*	*P*
rs79958549					
GG	265(80.30%)	312(94.55%)	0.15	1.00(reference)	
GA	39(11.82%)	17(5.15%)		2.70(1.49–4.89)	< 0.01
AA	26(7.88%)	1(0.30%)		2.10(4.13–2.18)	< 0.01
GA + AA vs. GG				4.25(2.46–7.35)	< 0.01
AA vs. GG + GA				2.01(1.63–2.09)	< 0.01
GG vs. AA				1.18(0.94–1.47)	0.17
G	569(86.21%)	641(97.12%)		1.00(reference)	
A	91(13.79%)	19(2.88%)		5.40(3.25–8.96)	< 0.01
rs139085828					
GG	281(85.15%)	314(95.15%)	0.09	1.00(reference)	
GA	29(8.79%)	15(4.55%)		2.16(1.14–4.11)	0.03
AA	20(6.06%)	1(0.30%)		2.02(1.55–2.12)	< 0.01
GA + AA vs. GG				1.60(1.31–1.83)	< 0.01
AA vs. GG + GA				1.96(1.51–2.06)	< 0.01
GG vs. AA				1.06(0.94–1.19)	0.36
G	591(89.55%)	643(97.42%)		1.00(reference)	
A	69(10.45%)	17(2.58%)		1.68(1.45–1.85)	< 0.01
rs201335371					
CC	186(56.36%)	121(36.67%)	0.09	1.00(reference)	
CT	105(31.82%)	145(43.94%)		0.47(0.34–0.66)	< 0.01
TT	39(11.82%)	64(19.39%)		0.40(0.25–0.63)	< 0.01
CT + TT vs. CC				0.45(0.33–0.61)	< 0.01
TT vs. CC + CT				0.56(0.36–0.86)	0.01
CC vs. TT				0.65(0.49–0.86)	< 0.01
C	477(72.27%)	387(58.64%)		1.00(reference)	
T	183(27.73%)	273(41.36%)		0.54(0.43–0.69)	< 0.01

*HWE* Hardy-Weinberg equilibrium, *OR* odds ratio, *CI* confidence interval

*Adjusted for age, gender, BMI, smoking, and drinking

### Analysis of linkage disequilibrium

Using Haploview 4.1 for linkage disequilibrium analysis, we found that SOX4 gene rs79958549, rs139085828, and rs201335371 loci formed a total of 5 haplotypes (Table 3). When the G-G-C haplotype was used as a reference, the A-A-C haplotype (OR = 5.14, 95% CI 2.45–10.57, *P* < 0.01) was associated with an increased risk of osteoporosis, while the G-G-T haplotype was significantly associated with a reduced risk of osteoporosis (OR = 0. 48, 95% CI 0.38–0.62, *P* <= 0.01).

**Table 3:** The correlation between the haplotypes of SOX4 gene rs79958549, rs139085828, and rs201335371 and the osteoporosis susceptibility

Haplotype #	Case(*n* = 330)	Control(*n* = 330)	OR (95% CI)	*P*
G-G-C	425(64.39%)	378(57.27%)	1.00(reference)	
A-G-T	7(1.06%)	1(0.15%)	6.23(0.76–50.84)	0.11
A-A-C	52(7.88%)	9(1.36%)	5.14(2.45–10.57)	< 0.01
A-A-T	10(1.52%)	9(1.36%)	0.99(0.40–2.46)	0.98
G-G-T	142(21.52%)	263(39.85%)	0.48(0.38–0.62)	< 0.01

^#^The haplotypes of SOX4 gene rs79958549, rs139085828, and rs201335371 loci

### Association of SOX4 gene rs79958549, rs139085828, and rs201335371 polymorphisms with clinical characteristics

The analysis results showed that there was no statistically significant difference in the factors of age, gender, BMI, smoking, and drinking among subjects with different genotypes of SOX4 rs79958549, rs139085828, and rs201335371 (*P* > 0.05). The BMD, *T*-score, and *Z*-score of subjects with different genotypes at the rs79958549, rs139085828, and rs201335371 loci of the SOX4 gene were significantly different (*P* < 0.01) (Tables 4, 5, and 6).

**Table 4:** Comparison of clinical characteristics among subjects with different genotypes at rs79958549 of SOX4 gene

	GG(*n* = 577)	GA(*n* = 56)	AA(*n* = 27)	*P*值
Age(years, mean ± SD)	65.51 ± 9.80	63.98 ± 10.35	65.44 ± 9.81	0.54
Gender [*n*(%)]				0.19
Male	300(51.99%)	22(39.29%)	14(51.85%)	
Female	277(48.01%)	34(60.71%)	13(48.15%)	
BMI(kg/m^2^, mean ± SD)	22.77 ± 2.21	22.68 ± 2.15	21.99 ± 2.48	0.20
Smoking				0.12
Yes	190(32.93%)	26(46.43%)	10(37.04%)	
No	387(67.07%)	30(53.57%)	17(62.96%)	
Drinking				0.13
Yes	213(36.92%)	19(33.93%)	15(55.56%)	
No	364(63.08%)	37(66.07%)	12(44.44%)	
BMD(g/m^2^)	0.88 ± 0.24	0.77 ± 0.17	0.74 ± 0.14	< 0.01
*T*-score	− 1.38 ± 1.84	− 1.61 ± 1.87	− 3.09 ± 0.88	< 0.01
*Z*-score	− 2.19 ± 1.39	− 3.23 ± 1.82	− 3.00 ± 1.37	< 0.01

*BMI* body mass index, *SD* Standard deviation

**Table 5:** Comparison of clinical characteristics among subjects with different genotypes at rs139085828 of SOX4 gene

	GG(*n* = 595)	GA(*n* = 44)	AA(*n* = 21)	*P*
Age(years, mean ± SD)	65.53 ± 9.82	63.07 ± 9.24	65.81 ± 11.38	0.27
Gender [*n*(%)]				0.84
Male	302(50.76%)	22(50.00%)	12(57.14%)	
Female	293(49.24%)	22(50.00%)	9(42.86%)	
BMI(kg/m2, mean ± SD)	22.76 ± 2.23	22.53 ± 2.08	22.19 ± 2.14	0.43
Smoking				0.82
Yes	204(34.29%)	16(36.36%)	6(28.57%)	
No	391(65.71%)	28(63.64%)	15(71.43%)	
Drinking				0.35
Yes	220(36.97%)	17(38.64%)	11(52.38%)	
No	375(63.03%)	27(61.36%)	10(47.62%)	
BMD(g/m^2^)	0.88 ± 0.24	0.78 ± 0.20	0.73 ± 0.15	< 0.01
*T*-score	− 1.42 ± 1.84	− 2.15 ± 1.84	− 3.15 ± 1.02	< 0.01
*Z*-score	− 2.25 ± 1.44	− 2.57 ± 1.58	− 3.50 ± 1.43	< 0.01

*BMI* body mass index, *SD* standard deviation

**Table 6:** Comparison of clinical characteristics among subjects with different genotypes at rs201335371 of SOX4 gene

	CC(*n* = 307)	CT(*n* = 250)	TT(*n* = 103)	*P*
Age(years, mean ± SD)	65.07 ± 9.74	64.87 ± 9.77	67.52 ± 10.11	0.06
Gender [*n*(%)]				0.64
Male	151(49.19%)	129(51.60%)	56(54.37%)	
Female	156(50.81%)	121(48.40%)	47(45.63%)	
BMI(kg/m^2^, mean ± SD)	22.65 ± 2.20	22.84 ± 2.25	22.68 ± 2.16	0.62
Smoking				0.62
Yes	108(35.18%)	87(34.80%)	31(30.10%)	
No	199(64.82%)	163(65.20%)	72(69.90%)	
Drinking				0.38
Yes	118(38.44%)	86(34.40%)	43(41.75%)	
No	189(61.56%)	164(65.60%)	60(58.25%)	
BMD(g/m^2^)	0.83 ± 0.23	0.89 ± 0.22	0.95 ± 0.27	< 0.01
*T*-score	− 1.92 ± 1.83	− 1.20 ± 1.80	− 1.13 ± 1.83	< 0.01
*Z*-score	− 2.50 ± 1.51	− 2.19 ± 1.50	− 2.05 ± 1.14	< 0.01

*BMI* body mass index, *SD* standard deviation, *BMD* bone mineral density

### Interaction of SOX4 rs79958549, rs139085828, and rs201335371 alleles with clinical characteristics

The multi-factor dimensionality reduction (MDR) was used to analyze the interaction between the alleles of SOX4 gene rs79958549, rs139085828, and rs201335371 and the clinical characteristics of the subjects’ age, gender, BMI, smoking conditions, and drinking conditions. The interaction among the factors of sex, smoking conditions, drinking conditions, rs79958549, and rs201335371 were the best model for osteoporosis prediction. The risk of osteoporosis in ‘high-risk combination’ was 2.74 times that of ‘low-risk combination’ (95% CI 1.01–7.43, *P* = 0.04, Table 7).

**Table 7:** MDR analysis of the interaction of SOX4 gene rs79958549, rs139085828, and rs201335371 alleles with the subjects’ age, gender, BMI, smoking conditions, and drinking conditions

Model	Training balanced accuracy	Training accuracy	OR (95% CI)	*χ*^2^	*P*
rs201335371	0.5985	0.5985	2.23(0.83–5.99)	2.57	0.11
rs79958549, rs201335371	0.6258	0.6258	2.80(1.03–7.58)	4.18	0.04
rs79958549, rs139085828, rs201335371	0.6348	0.6288	2.87(1.06–7.80)	4.38	0.04
Smoking, drinking, rs79958549, rs201335371	0.6517	0.5864	2.02(0.76–5.42)	1.99	0.16
Gender, smoking, drinking, rs79958549, rs201335371	0.6704	0.6227	2.74(1.01–7.43)	3.99	0.04
Gender, smoking, drinking, rs79958549, rs139085828, rs201335371	0.6916	0.5985	2.24(0.83–6.02)	2.58	0.11
Age, gender, smoking, alcohol, rs79958549, rs139085828, rs201335371	0.7114	0.5742	1.82(0.69–4.83)	1.46	0.23
Age, gender, BMI, smoking, alcohol, rs79958549, rs139085828, rs201335371	0.7348	0.5591	1.62(0.61–4.31)	0.93	0.33

*OR* odds ratio, *CI* confidence interval, *BMI* body mass index

### Multiple logistic regression analysis

A number of variables were tested in this study to determine whether they were independent risk factors for osteoporosis. As shown in Table 8. The risk factors for osteoporosis were BMD (OR = 5.85, 95% CI 2.88–8.94, *P* < 0.01), *T*-score (OR = 8.54, 95% CI 5.66–10.49, *P* < 0.01), *Z*-score (OR = 3.77, 95% CI 2.15–8.50, *P* < 0.01), rs79958549 SNP (OR = 6.92, 95% CI 3.58–8.93, *P* < 0.01), and rs139085828 SNP (OR = 2.36, 95% CI 1.85–4.27, *P* < 0.01); the protective factor was rs201335371SNP (OR = 0.48, 95% CI 0.32–0.75, *P* < 0.01) (Table 8).

**Table 8:** Logistic regression analysis for identifying risk factors of osteoporosis

Variables	*B*	OR (95%CI)	*P* value
Age	− 0.01	0.99(0.79–1.17)	0.12
Gender	− 0.19	0.83(0.55–1.44)	0.85
BMI	0.04	1.04(0.85–1.77)	0.63
Smoking	0.10	1.11(0.94–2.01)	0.55
Drinking	− 0.98	0.83(0.22–1.55)	0.44
BMD	4.56	5.85(2.88–8.94)	< 0.01
*T*-score	14.55	8.54(5.66–10.49)	< 0.01
*Z*-score	2.64	3.77(2.15–8.50)	< 0.01
rs79958549	1.95	6.92(3.58–8.93)	< 0.01
rs139085828	0.86	2.36(1.85–4.27)	< 0.01
rs201335371	− 0.73	0.48(0.32–0.75)	< 0.01

*BMI* body mass index, *BMD* bone mineral density

## Discussion

By conducting a case-control study, we found that the SOX4 gene rs79958549 A allele, rs139085828 A allele, and rs201335371 C allele were significantly associated with an increased risk of osteoporosis. We also found that the A-A-C haplotype formed by rs79958549, rs139085828, and rs201335371 of SOX4 gene was associated with an increased risk of osteoporosis, and the G-G-T haplotype was associated with a reduced risk of osteoporosis. The MDR analysis indicated that the interaction between gender, smoking, drinking, rs79958549, and rs201335371 was significantly related to the risk of osteoporosis. Multiple logistic regression analysis revealed that the risk factors for osteoporosis were BMD (OR = 5.85, 95% CI 2.88–8.94, *P* < 0.01), *T*-score (OR = 8.54, 95% CI 5.66–10.49, *P* < 0.01), *Z*-score (OR = 3.77, 95% CI 2.15–8.50, *P* < 0.01), rs79958549 SNP (OR = 6.92, 95% CI 3.58–8.93, *P* < 0.01), and rs139085828 SNP (OR = 2.36, 95% CI 1.85–4.27, *P* < 0.01); the protective factor was rs201335371SNP (OR = 0.48, 95% CI 0.32–0.75, *P* < 0.01). The rs79958549, rs139085828, and rs201335371 loci of the SOX4 gene and their interaction with environmental factors were associated with the risk of osteoporosis.

In recent years, the focus of relevant research has been mainly on the correlation between osteoporosis-related genes and osteoporosis [14, 15], and at the same time, the purpose of curing the disease by modifying genetic material [16]. There are many SNP loci in the coding region of the SOX4 gene, and the MAF of most SNP loci was below 0.01. In the 1000 genomes database, the MAF of rs79958549 was 0.024, that of rs139085828 was 0.0102, and that of rs201335371 was 0.4320, all above 0.01. In the control group, the MAF of rs79958549 was 0.0288, that of rs139085828 was 0.0258, and that of rs201335371 was 0.4136, close to the data in the 1000 genomes database, indicating that the population selected in this study was representative. In addition, using the allele frequency of rs79958549 was used as a reference, we calculated the minimum sample size required for the case and the control group was 97 and 97 cases, respectively; using the allele frequency of rs139085828 as a reference, the minimum sample size required was 150 cases and 150 cases respectively; and using the allele frequency of rs201335371 as a reference, the minimum sample size required was 187 cases and 187 cases. All are lower than the sample size in this study, which indicated that the results are relatively objective.

In this study, the subjects with the GA or AA genotype at rs79958549 had a higher risk of osteoporosis than those with the GG genotype. After adjusting for age, gender, BMI, smoking, drinking, and other factors, the rs79958549 A allele carriers had a 5.40 times higher risk than the G allele carriers, suggesting that rs79958549 A allele was associated with an increased risk of osteoporosis. For rs139085828, the GA and AA genotypes were associated with an increased risk of osteoporosis, and the A allele carriers had a higher risk of osteoporosis than the G allele carriers. For rs201335371, the carriers of the CT and TT genotypes had a lower risk of osteoporosis than the carriers of the CC genotype, and the T allele is a protective gene for osteoporosis. At present, there is no research focusing on the correlation between these SNP loci and diseases.

Many researchers focused on the correlation between the SOX4 gene and tumors, and obtained some research findings. Increased SOX4 expression often inhibits apoptosis and increases cell invasion and metastasis, and drug resistance in most tumors, such as oral squamous cell carcinoma [17], lung cancer [18], breast cancer [19], gastric cancer [20], hepatocellular carcinoma [21], colorectal cancer [22], endometrial cancer [23], bladder cancer [11], and prostate cancer [24]. The latest research has shown that the SOX4 can participate in the pathological changes of osteoarthritis cartilage by regulating ADAMTS4 and ADAMTS5 [25]. It was found in a mouse model that SOX4 mRNA expression was increased in the cartilage of the osteoarthritis patients, resulting in articular cartilage destruction through adenovirus infection. However, the specific mechanism of SOX4 in the development of osteoporosis remains unclear. Some studies have shown reduced bone mass and bone formation and impaired osteoblast development in SOX4 heterozygous mice, indicating the significant role of SOX4 in bone formation and resorption.

It is acknowledged that the occurrence of osteoporosis is closely related to genetic and environmental factors [26]. MDR is a new method developed in recent years to analyze interactions, and its greatest advantage is the ability to simultaneously detect and analyze the combined effects of multiple factors influencing the disease. It does not consider main effects when analyzing interactions between factors and levels. Therefore, it can still detect higher-order interactions when the potential main effects are not statistically significant. MDR can only detect interactions, but it cannot detect main effects when they are significant. In this case, with the help of logistic regression, after first detecting the interaction with MDR, the interaction term is forced into logistic regression for main effect and interaction effect analysis. Our analysis showed that the interaction among sex, smoking, drinking, rs79958549, and rs201335371 was of great significance for the prediction of osteoporosis risk. We found that the ‘high-risk combination’ of these factors was 2.74 times more likely to develop osteoporosis than the ‘low-risk combination’, which, combined with the results of logistic regression analysis, suggests that the interaction between the SOX4 gene and the environment is of great value for osteoporosis risk prediction.

The study provides a new idea for the prevention and treatment of osteoporosis. The findings suggesting that studies on osteoporosis-related gene polymorphisms and their interaction with environmental factors may contribute to new directions for the prevention and treatment of osteoporosis. However, this study has some deficiencies. First, the molecular mechanism of the SOX4 gene rs79958549, rs139085828, and rs201335371 related to osteoporosis risk needs to be further studied. Whether these SNP loci are located at the binding sites of microRNAs to the SOX4 gene needs to be predicted by bioinformatics tools. Second, the correlation between the SOX4 gene rs79958549, rs139085828, rs201335371 loci and the expression levels of SOX4 needs further study. Furthermore, the potential role of SOX4 in the occurrence and development of osteoporosis needs to be confirmed by both in vitro and in vivo research.

## Conclusion

We found that the SOX4 gene SNPs rs79958549, rs139085828, and rs201335371 are significantly associated with osteoporosis risk. Also, the interaction between sex, smoking, drinking, rs79958549, and rs201335371 is of great significance in osteoporosis risk prediction.

## Data Availability

The datasets used and/or analyzed during the current study are available from the corresponding author on reasonable request.

## Abbreviations

SOX4: SRY-related high-mobility-group box gene 4
UTR: Untranslated region
SNP: Single nucleotide polymorphism
MDR: Multi-factor dimensionality reduction
SRY: Sex determining region Y
MAF: Minor allele frequency
BMD: Bone mineral density
DXA: Dual-energy X-ray absorptiometry
CI: Confidence interval
BMI: Body mass index

## References

[1] Aspray TJ, Hill TR (2019). Osteoporosis and the ageing skeleton. Subcell Biochem.

[2] Lane NE (2006). Epidemiology, etiology, and diagnosis of osteoporosis. Am J Obstet Gynecol.

[3] Miller PD (2016). Management of severe osteoporosis. Expert Opin Pharmacother.

[4] Kanis JA, Cooper C, Rizzoli R, Reginster JY, Scientific Advisory Board of the European Society for C, Economic Aspects of O, the Committees of Scientific A, National Societies of the International Osteoporosis F (2019). European guidance for the diagnosis and management of osteoporosis in postmenopausal women. Osteoporos Int.

[5] Cosman F, de Beur SJ, LeBoff MS, Lewiecki EM, Tanner B, Randall S, Lindsay R, National Osteoporosis F (2014). Clinician's Guide to Prevention and Treatment of Osteoporosis. Osteoporos Int.

[6] Kim BJ, Lee YS, Lee SY, Baek WY, Choi YJ, Moon SA, Lee SH, Kim JE, Chang EJ, Kim EY, Yoon J, Kim SW, Ryu SH, Lee SK, Lorenzo JA, Ahn SH, Kim H, Lee KU, Kim GS, Koh JM (2018). Osteoclast-secreted SLIT3 coordinates bone resorption and formation. J Clin Invest.

[7] Li G, Zhang L, Wang L, Yuan G, Dai K, Pei J, Hao Y (2018). Dual modulation of bone formation and resorption with zoledronic acid-loaded biodegradable magnesium alloy implants improves osteoporotic fracture healing: An in vitro and in vivo study. Acta Biomater.

[8] Sinclair AH, Berta P, Palmer MS, Hawkins JR, Griffiths BL, Smith MJ, Foster JW, Frischauf AM, Lovell-Badge R, Goodfellow PN (1990). A gene from the human sex-determining region encodes a protein with homology to a conserved DNA-binding motif. Nature.

[9] Vervoort SJ, de Jong OG, Roukens MG, Frederiks CL, Vermeulen JF, Lourenco AR, et al. Global transcriptional analysis identifies a novel role for SOX4 in tumor-induced angiogenesis. Elife 7. 2018;7. 10.7554/eLife.27706. 10.7554/eLife.27706 PMC6277201 30507376

[10] Hoser M, Baader SL, Bosl MR, Ihmer A, Wegner M, Sock E (2007). Prolonged glial expression of Sox4 in the CNS leads to architectural cerebellar defects and ataxia. J Neurosci.

[11] Moran JD, Kim HH, Li Z, Moreno CS (2019). SOX4 regulates invasion of bladder cancer cells via repression of WNT5a. Int J Oncol.

[12] Nissen-Meyer LS, Jemtland R, Gautvik VT, Pedersen ME, Paro R, Fortunati D, Pierroz DD, Stadelmann VA, Reppe S, Reinholt FP, Del Fattore A, Rucci N, Teti A, Ferrari S, Gautvik KM (2007). Osteopenia, decreased bone formation and impaired osteoblast development in Sox4 heterozygous mice. J Cell Sci.

[13] Duncan EL, Danoy P, Kemp JP, Leo PJ, McCloskey E, Nicholson GC, Eastell R, Prince RL, Eisman JA, Jones G, Sambrook PN, Reid IR, Dennison EM, Wark J, Richards JB, Uitterlinden AG, Spector TD, Esapa C, Cox RD, Brown SD, Thakker RV, Addison KA, Bradbury LA, Center JR, Cooper C, Cremin C, Estrada K, Felsenberg D, Gluer CC, Hadler J, Henry MJ, Hofman A, Kotowicz MA, Makovey J, Nguyen SC, Nguyen TV, Pasco JA, Pryce K, Reid DM, Rivadeneira F, Roux C, Stefansson K, Styrkarsdottir U, Thorleifsson G, Tichawangana R, Evans DM, Brown MA (2011). Genome-wide association study using extreme truncate selection identifies novel genes affecting bone mineral density and fracture risk. PLoS Genet.

[14] Scimeca M, Salustri A, Bonanno E, Nardozi D, Rao C, Piccirilli E, Feola M, Tancredi V, Rinaldi A, Iolascon G, Orlandi A, Gasbarra E, Maffulli N, Brandi ML, Tarantino U (2017). Impairment of PTX3 expression in osteoblasts: a key element for osteoporosis. Cell Death Dis.

[15] Conti V, Russomanno G, Corbi G, Toro G, Simeon V, Filippelli W, Ferrara N, Grimaldi M, D'Argenio V, Maffulli N, Filippelli A (2015). A polymorphism at the translation start site of the vitamin D receptor gene is associated with the response to anti-osteoporotic therapy in postmenopausal women from southern Italy. Int J Mol Sci.

[16] Aicale R, Tarantino D, Maccauro G, Peretti GM, Maffulli N, Xix Congresso Nazionale S.I.C.O.O.P. Societa' Italiana Chirurghi Ortopedici Dell'Ospedalita' Privata A (2019). Genetics in orthopaedic practice. J Biol Regul Homeost Agents.

[17] Yoon TM, Kim SA, Cho WS, Lee DH, Lee JK, Park YL, Lee KH, Lee JH, Kweon SS, Chung IJ, Lim SC, Joo YE (2015). SOX4 expression is associated with treatment failure and chemoradioresistance in oral squamous cell carcinoma. BMC Cancer.

[18] Hu B, Zhang H, Wang Z, Zhang F, Wei H, Li L (2017). LncRNA CCAT1/miR-130a-3p axis increases cisplatin resistance in non-small-cell lung cancer cell line by targeting SOX4. Cancer Biol Ther.

[19] Sharma S, Nagpal N, Ghosh PC, Kulshreshtha R (2017). P53-miR-191-SOX4 regulatory loop affects apoptosis in breast cancer. RNA.

[20] Ding L, Zhao Y, Dang S, Wang Y, Li X, Yu X, Li Z, Wei J, Liu M, Li G (2019). Circular RNA circ-DONSON facilitates gastric cancer growth and invasion via NURF complex dependent activation of transcription factor SOX4. Mol Cancer.

[21] Hur W, Rhim H, Jung CK, Kim JD, Bae SH, Jang JW, Yang JM, Oh ST, Kim DG, Wang HJ, Lee SB, Yoon SK (2010). SOX4 overexpression regulates the p53-mediated apoptosis in hepatocellular carcinoma: clinical implication and functional analysis in vitro. Carcinogenesis.

[22] Lei X, Li L, Duan X. Long non-coding RNA ABHD11-AS1 promotes colorectal cancer development through regulation of miR-133a/SOX4 axis. Biosci Rep. 2018;38(6). 10.1042/BSR20181386. 10.1042/BSR20181386 PMC6294614 30429229

[23] Du Q, Liu J, Zhang X, Zhang X, Zhu H, Wei M, Wang S (2018). Propofol inhibits proliferation, migration, and invasion but promotes apoptosis by regulation of Sox4 in endometrial cancer cells. Braz J Med Biol Res.

[24] Liu H, Wu Z, Zhou H, Cai W, Li X, Hu J, Gao L, Feng T, Wang L, Peng X, Qi M, Liu L, Han B (2019). The SOX4/miR-17-92/RB1 axis promotes prostate cancer progression. Neoplasia.

[25] Takahata Y, Nakamura E, Hata K, Wakabayashi M, Murakami T, Wakamori K, Yoshikawa H, Matsuda A, Fukui N, Nishimura R (2019). Sox4 is involved in osteoarthritic cartilage deterioration through induction of ADAMTS4 and ADAMTS5. FASEB J.

[26] Ralston SH, de Crombrugghe B (2006). Genetic regulation of bone mass and susceptibility to osteoporosis. Genes Dev.

